# Novel *CHM* mutations identified in Chinese families with Choroideremia

**DOI:** 10.1038/srep35360

**Published:** 2016-10-14

**Authors:** Xue-Bi Cai, Xiu-Feng Huang, Yi Tong, Qin-Kang Lu, Zi-Bing Jin

**Affiliations:** 1 The Eye Hospital of Wenzhou Medical University, The State Key Laboratory Cultivation Base and Key Laboratory of Vision Science, Ministry of Health, Wenzhou 325027, China; 2 Fuzhou Southeastern Eye Hospital, Fuzhou, 350000, China; 3 Department of Ophthalmology, Yinzhou People’s Hospital, Medical School of Ningbo University, Ningbo, 315040, China

## Abstract

Choroideremia is a bilateral and progressive X-linked inherited disease characterized by widespread chorioretinal atrophy with relative sparing of the macular region. It is caused by mutations in the ubiquitously expressed *CHM* gene, which lead to the absence of the Rab escort protein 1 (REP-1), resulting in prenylation deficiency. Typical fundus appearances for choroideremia were found in 3 probands from three unrelated Chinese families in our study. We firstly used the targeted exome sequencing (TES) technology to detect mutations in *CHM* gene. Based on an established filtering strategy of data analyses, along with confirmation by co-segregation, a previously reported mutation (c.1584_1587del TGTT, p.V529Hfs*7) was identified in one family, while two novel mutations (c.227_232delinsTGTCATTTCA, p.Q76Lfs*7; c.710dupA, p.Y237_S238delinsX) were identified in the other two families. These findings not only expands the currently limited spectrum of Chinese disease-causing variants in *CHM* gene, but also increases our understanding of the phenotypic and genotypic correlations of choroideremia, and may potentially lead to improved genetic counseling and specific treatment for families with choroideremia as well.

Choroideremia (CHM; MIM 303100) is an X-linked recessive eye disorder, with the incidence ranging from 1:50,000 to 1:100,000^1^,^2^. It commonly manifests as a progressive-inherited chorioretinal degenerative disease characterized by a bilateral, diffuse atrophy of the choriocapillaris, retinal pigment epithelium (RPE), and photoreceptors^3^. Affected males develop night blindness in late childhood, which progresses with a gradual loss of peripheral vision. Normally, their vision deterioration can reach legal blindness by middle age. Female carriers generally have normal vision, visual fields, color vision, and electroretinograms (ERGs); however, due to random X-inactivation (lyonization), patchy areas of chorioretinal degeneration can occur in women^4^. In both men and women, central vision is typically maintained until late in the progression of the disease.

In patients with choroideremia, diagnoses are confirmed with the identification of mutations in the *CHM* gene, which is mapped to chromosome Xq21.2^3^. The *CHM* gene spans over 180 Kb on the X chromosome, and is expressed in many tissue types including retinal photoreceptor, choroid, RPE, and lymphocyte tissues. The mRNA consists of 15 exons that are conserved in transcription. All of the conserved exons, except exon 15 (3,642 bp), are fewer than 400 bp long^5^. Furthermore, the absence of Rab escort protein-1 (REP-1) encoded by *CHM*, which results in prenylation deficiency, has been verified as the cause of retinal degeneration in choroideremia^6^. As an intracellular protein of 653 amino acids and the A component of Rab geranylgeranyltransferase (GGTase), REP-1 plays an important role in the post-translational Isoprenyl modification of Rab proteins as well as in assisting the intracellular vesicular trafficking process^7^,^8^. In all tissues, with the exception of the eye, the loss of functioning REP-1 appears to be compensated by REP-2^9^. Consequently, REP-1 function seems to be a vital factor for the function of the retinal pigment epithelium and photoreceptors.

Almost all reported cases of choroideremia so far have been attributed to functionally null mutations combined with the slow rate of degeneration and small size of the CHM protein coding sequence, which make gene therapy with adeno-associated viral (AAV) vectors an appealing treatment strategy. The subsequent preclinical data for the effects of the vector (AAV2.REP1) in restoring prenylation activity to human choroideremia fibroblasts and initial findings from a phase 1/2 clinical trial have been described^10^,^11^.

In published literatures, a wealth of disease-causing mutations have been reported, including nonsense mutations, missense mutations, frameshifts, splice site defects, gross insertions, small deletions, and deletion of the entire *CHM* gene. More than 100 different pathogenic CHM mutations are currently documented in the RetinoGenetics^12^. In spite of the large existing spectrum of disease-causing variants in *CHM* gene, the investigations of Chinese patients with choroideremia are rather limited. We herein describe the phenotype and genotype of members in three Chinese families with choroideremia, and disclose the detection of novel mutations of the *CHM* gene firstly with the targeted exome sequencing (TES) technology.

## Materials and Methods

### Subjects and phenotyping

The study protocol was in accordance with the tenets of the Declaration of Helsinki and approved by the Ethics Committee of The Eye Hospital of Wenzhou Medical University. Informed consent was obtained from each participant. Three probands and two of their family members were enrolled in this study. Each of them underwent a comprehensive ophthalmologic examination comprising best-corrected visual acuity (BCVA), slit-lamp biomicroscopy, and color vision screening, which was supplemented with a series of imaging studies including fundus photography and optical coherence tomography (OCT). A panel of ophthalmologists and optometrists reviewed all ocular findings, and confirmed the clinical diagnosis of choroideremia in affected individuals. We contacted all of the genetically related family members, and obtained a detailed family history from each of that were recruited. Moreover, the five participants donated their blood for molecular studies. Physical examinations were performed to exclude systemic diseases, and no other remarkable diseases were found in the subjects’ medical histories.

### Library preparation and Targeted exome sequencing

Genomic DNA was extracted from peripheral blood using standard organic extraction procedures. The DNA samples were quantified using a Nanodrop 2000 (Thermal Fisher Scientific, DE), and stored below −20 °C until experimental use. Libraries were prepared in the light of Illumina standard protocol. Briefly, a minimum of 3 μg of the DNA was sheared into 350- to 400-bp fragments. The fragmented DNA was end-repaired, a single adenine base was added to 3′ end, Illumina adapters were then ligated to the fragments, and the final products were PCR amplified. The probes, aiming to be hybridized in solution with the target library for TES experiments, were designed to potentially tile along 164 known causative inherited retinal dystrophy (IRD) genes, including one choroideremia-related gene. Afterwards, Illumina sequencing was performed in according to a previous study^13^.

### Bioinformatics analysis

Sequencing reads were aligned to human hg19 reference using BWA (http://bio-bwa.sourceforge.net/), and afterwards, base quality recalibration and local realignment were conducted using the GATK program (http://www.broadinstitute.org/gsa/wiki/index.php/Home_Page)^14^,^15^. To filter out common SNPs and InDels, with minor allele frequency (MAF) > 0.01, the Exome-assistant program (http://122.228.158.106/exomeassistant) was then used for annotation of all identified variants. MagicViewer was applied to view the short read alignment and ascertain the candidate SNPs and InDels. Missense variants were assessed with PolyPhen-2 (http://genetics.bwh.harvard.edu/pph2/) and SIFT (http://sift.jcvi.org/www/SIFT_enst_submit.html), while the splice-site effect was predicted using the BDGP (http://www.fruitfly.org/seq_tools/splice.html) and ASSP programs (http://wangcomputing.com/assp/index.html).

### PCR and direct Sanger sequencing

The DNA samples from the 5 participants in this study, as well as 200 unrelated Chinese control individuals, were analyzed by DNA sequencing. All 15 coding exons of the *CHM* gene were amplified by PCR. The reagents used for a 50 μl reaction were as follows: 25 μl of 2 × Power Taq PCR MasterMix (BIOTEKE CORPORATION), 2.5 μl each of 10 μM forward and reverse primer, 100 ng of genomic DNA template, and sterile H_2_O. Amplification was performed in a thermal cycler (Applied Biosystems Veriti 96-Well) with the following PCR protocol: initial DNA denaturation occurred at 95 °C for 5 min, 35 cycles of 94 °C for 30 s, 55 °C for 30 s, and 72 °C for 60s, and a final extension phase at 72 °C for 7 min. Afterward, the temperature was held at 4 °C. PCR products were subjected to agarose gel electrophoresis, excised from the gel, and subsequently sequenced. The sequencing results were analyzed using FinchTV (version 1.4.0), and compared with the NCBI database reference sequences. Sanger sequencing and co-segregation analyses in the family member samples confirmed the candidate variants.

## Results

### Clinical features

**Figure 1:**
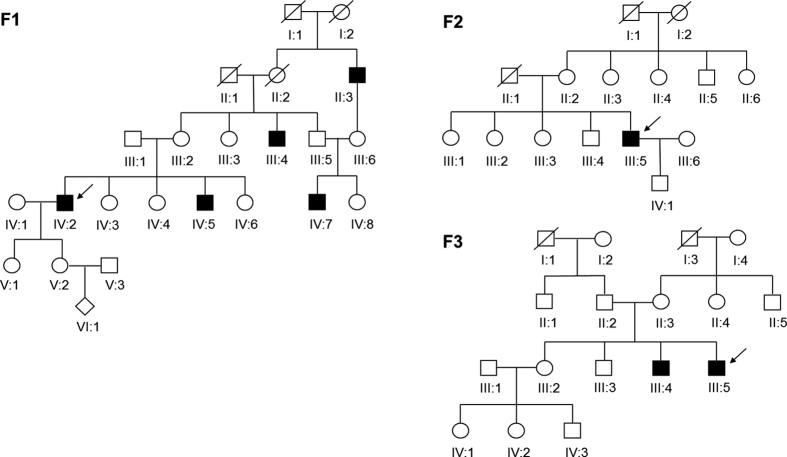
Pedigrees of three Chinese families with choroideremia. Squares and circles indicate males and females, respectively. Rhombus refers to the infant with unknown gender. Deceased family members are represented with a diagonal line, and darkened symbols represent the affected members. The patients below the arrow are the probands.

**Figure 2:**
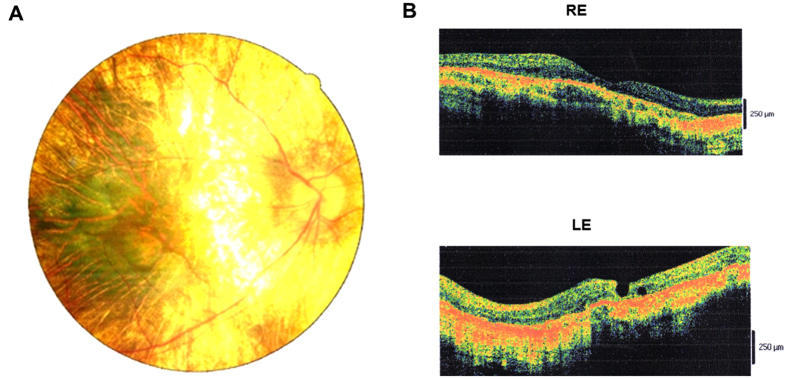
Imaging studies of the proband F1:IV:2. (**A**) Fundus photography showing widespread confluent areas of nummular RPE and choriocapillaris atrophy in the midperiphery and in the paramacular area, sparing only the centermost macular region. (**B**) OCT images showing reduction of retinal thickness and anomalous reflectivity corresponds well with relative chorioretinal atrophy. RE, right eye; LE, left eye.

The inheritance patterns of the three Chinese families were all X-linked, as indicated by the familial pedigrees (Fig. 1). The proband F1:IV:2 complained of a long history of night blindness, a progressive decrease in his peripheral vision of each eye, and difficulty with color discrimination. He had normal corneas and anterior chambers, while his lenses were a little turbid (+), which revealed a mild cataract condition. Additionally, he was affected with metamorphopsia. Color vision screening with Ishihara color plates disclosed his limited ability only to identify the test plates in each eye. Fundus exam showed a scalloped border of atrophic changes with easily visible radial choroidal vessels near the macula. There was a residual island of healthy tissue in the central macula and fovea. The optic disc was normal with peripapillary pigment atrophy and the retinal vessels were attenuated (Fig. 2A). OCT images presented a central macular thinning of the retinal outer layers, as well as a discordant reflection pattern within the RPE layer and the choroicapillary layer of each eye, and a discontinuity was found in the retinal neurosensory layer of the left eye (Fig. 2B). The clinical characterizations of the other two probands F2:III:5 and F3:III:5, were very similar to that of the proband F1:IV:2. So was that of the affected male F3:III:4. The female carrier F1:V:2, however, seemed to be asymptomatic. The results for the optometry check of the five participants were shown in Table 1.

### Candidate mutations identified by TES

We designed a capture panel to enrich the target DNA by selecting 164 known IRD-related genes from RetNet and OMIM. The enriched DNA was then sent for TES. More than 96.9% of the targeted disease gene regions were sequenced, with an average sequencing depth of 104.38. A total number of 650 variants were identified in the samples. Among them, there were 386 non-synonymous variants, including missense, nonsense, and splicing variants. This number was narrowed down to 46 variants by excluding variants reported in dbSNP137, Exome Variant Server, and ExAC that had minor allele frequencies of (MAF) > 0.01. For missense variants, computational prediction by PolyPhen and SIFT as well as imposing restriction such as the consistency of genetic transmission mode were applied to further reduce the number of candidate mutations. The remaining variants were likely to cause the disease in patients and were further subjected to validation by Sanger sequencing.

### Confirmation and co-segregation testing

**Figure 3:**
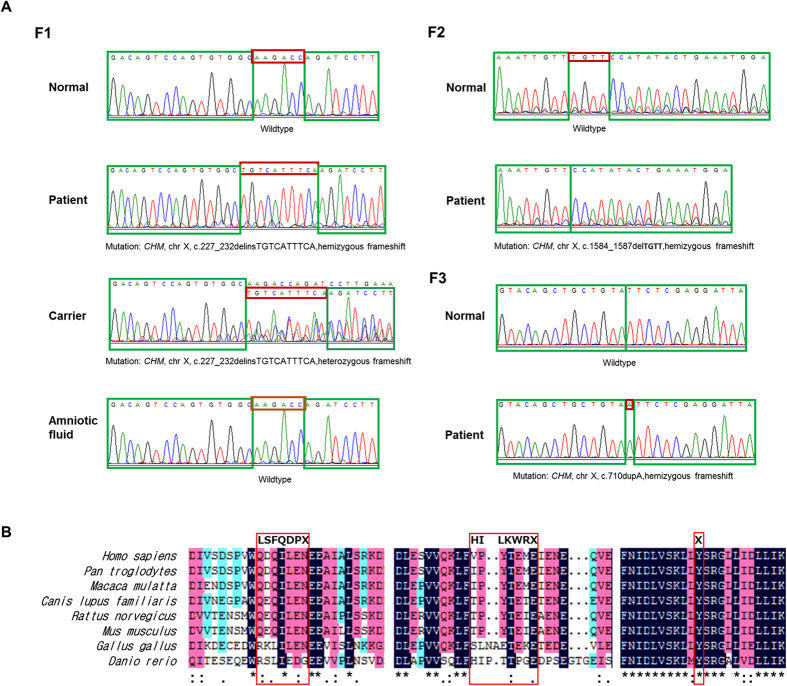
Mutational analyses of the three Chinese families. (**A**) Sequencing results of the family members recruited. Compared to the controls, the male patient F1:IV:2 and the female carrier F1:V:2 from family 1 have an identical 6-bp deletion with a 10-bp insertion mutation, the male patient F2:V:5 from family 2 has a 4-bp deletion mutation, and the two male patients F3:III:5 and F3:III:4 from family 3 have the same duplication mutation. (**B**) Multiple sequence alignment of CHM polypeptides of different species showing the conserved amino acid residues. The amino acid residues highlighted in bold in the red boxes are the residues after frameshift mutation.

After a comprehensive screening for mutations by Sanger sequencing and co-segregation analyses of the three Chinese pedigrees, three frameshift mutations in *CHM* gene were detected, none of which occurred in the 200 controls (Fig. 3A and Supp. Fig. 1). In family 1, the proband F1:IV:2 harbored a hemizygous mutation (c.227_232delinsTGTCATTTCA. p.Q76Lfs*7) within exon 4, which had not been reported and thus was ascertained to be novel. However, his pregnant daughter F1:V:2 was heterozygous for the mutation, while the amniotic fluid was finally confirmed to be wildtype, showing she was carrying a healthy infant. In family 2, a previously reported mutation (c.1584_1587del TGTT. p.V529Hfs*7) within exon 13 was detected^16^,^17^,^18^. In family 3, the proband F3:III:5 and his brother F3:III:4 both harbored a novel hemizygous mutation (c.710dupA, p.Y237_S238delinsX) within exon 6. The three mutations were predicted to stop the open reading frame at codon 82, codon 535, and codon 237, respectively, thereby generating three different prematurely truncated *CHM* proteins (Fig. 3B).

## Discussion

Choroideremia occurs in about 4% of the blind population, and has become the second leading cause of hereditary and progressive bilateral night blindness after retinitis pigmentosa. In this study, we reported three Chinese families with choroideremia and shed light on the phenotypic and genotypic defects present in the family members.

The probands showed severe degeneration in their visual acuity when observed in their ophthalmic examinations, and widespread chorioretinal atrophies were seen using fundoscopy and optical coherence tomography scans. A notable difference, in comparison with other retinal degenerative diseases, is that all target tissue layers, including the choroid, RPE, and photoreceptors, are continually shrinking in choroideremia^11^. Due to the deficiency in the choroid and the fragility of the blood-retina barrier, the affected eyes tend to be vulnerable to other various diseases, which results in direct or indirect damages^19^. Patients with choroideremia have been reported to have complications involving cataracts^20^, recurrent uveitis^21^, and cystic macular edema^22^. Clinical findings from the affected male F1:IV:2 in our study included poor visual acuity, cataracts, metamorphopsia, and decreased color vision, in addition to the chorioretinal atrophy consistent with the choroideremia diagnosis. Despite these clinical findings, the female carrier F1:V:2 was asymptomatic.

**Figure 4:**
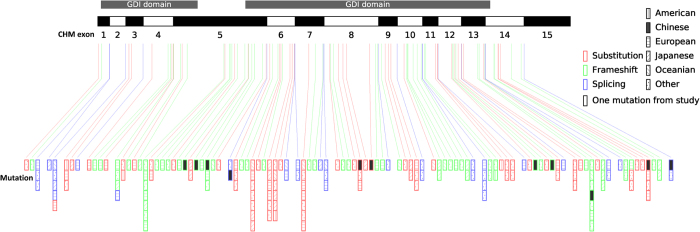
Schematic diagram of the mutations in the CHM gene. The black and white bars of various lengths with numbers below them represent different exons. The longitudinal bars indicate different types of mutations within different exons and flanking introns, with the red bars showing substitution mutations, the green bars showing frameshift mutations, and the purple bars showing splicing mutations. The number of the colored longitudinal bars corresponds with the number of reported mutations found in multiple ethnic groups. The mutations occur in different ethnicities are noted by the vertical line, black patch, horizontal lines, left diagonals, right diagonals and black dots inside the bars, respectively representing American, Chinese, European, Japanese, Oceanian, and other nationalities. The gray transverse bars in the top tier indicate the GDI domains.

Mutations in the *CHM* gene cause choroideremia in multiple ethnic groups. Previous studies found that cases of choroideremia had been mostly reported in European and Japanese families, but reported *CHM* mutations from Chinese families have been limited (Fig. 4). Yip *et al.*^23^ reported five mutations in five separate Chinese families, Zhou *et al.*^24^ detected three mutations in three mainland Chinese families, Lin *et al.*^19^ identified two mutations in two Chinese families, and Guo *et al.*^25^ disclosed one frameshift mutation in a Chinese family. From our experimental analyses of three unrelated Chinese families, we expand the spectrum of choroideremia-related mutations by adding two novel mutations (c.227_232delinsTGTCATTTCA, p.Q76Lfs*7; c.710dupA, p.Y237_S238delinsX) and a previously reported mutation (c.1584_1587del TGTT, p.V529Hfs*7) to the existing disease-causing variants in *CHM* gene.

In this work, candidate variants were only considered pathogenic when they satisfied following criteria: (1) missense mutations were positioned in the amino-acid conserved region across species, (2) splice-site variations fulfilled the GT-AT rules, (3) stop/frameshift variants were present, and (4) the mutations were predicted to be damaging or disease-causing by no less than two of the bioinformatic programs (PolyPhen-2, SIFT, MutationTaster, and ASSP)^26^. The novel mutations found in family 1 and family 3 of our study both met the third criterion, and were further verified to cause the premature translation of the protein. As a result, they were critically confirmed to be pathogenic equivalents to the known mutation detected in family 2 (Table 2). Most of the *CHM* mutations result in absence of REP-1 due to premature termination codons (PTCs) and degradation of the inappropriately folded protein or truncated mRNA^27^,^28^. Our data demonstrated three different types of truncations in the *CHM* gene, producing a truncated REP-1 protein of 81 aa, 534 aa, and 236 aa, respectively. The truncated REP-1 protein was assumed to be degraded enzymatically *in vivo* in the affected members of these CHM families.

In summary, our study uncovered the clinical and genetic characteristics of three Chinese families with choroideremia. The primary methods used to identify disease-causative genes are currently positional cloning and linkage analysis. Nevertheless, there are tremendous limitations within the conventional strategies. Recently, targeted exome sequencing (TES) technology has become an efficient for exploring disease-related genes^29^. We firstly used the TES approach to successfully identify mutations in CHM from pedigrees with choroideremia, including two novel mutations as well as a known causative mutation. Considering the developing retinal gene therapy for choroideremia, this work will not only increase our understanding of the genetic etiology of choroideremia, but may potentially lead to improved genetic counseling and specific treatment for families with choroideremia as well.

## Additional Information

**How to cite this article**: Cai, X.-B. *et al.* Novel *CHM* mutations identified in Chinese families with Choroideremia. *Sci. Rep.* **6**, 35360; doi: 10.1038/srep35360 (2016).

## Supplementary Material

Supplementary Information

## Figures and Tables

**Table 1:** Visual acuity of the five participants in this study.

Subjects	Age/Onset		Distant vision	Near vision	Auto-Ref	Sub-Ref	BCVA	PD(mm)
F1:IV:2	46/10	OD	0.4	0.4/20 cm	−2.50/–0.75 × 25	−2.50/–0.75 × 30	0.8	69
		OS	0.05	0.05/20 cm	−2.50/–1.75 × 165	−2.50/–1.75 × 170	0.6	
F1:V:2	25/−	OD	0.8	0.8/20 cm	−1.25/–0.75 × 160	−1.25/–0.75 × 155	1.2	61
		OS	0.8	0.8/20 cm	−1.00/–1.00 × 8	−1.00/–1.00 × 15	1.2	
F2:V:5	44/8	OD	LP	LP	−5.00/–4.50 × 179	LP	LP	68
		OS	LP	LP	−6.75/–3.50 × 107	LP	LP	
F3:III:4	28/12	OD	0.15	0.16/20 cm	−3.50/–1.25 × 99	−3.25/–1.00 × 100	1.0	63
		OS	0.12	0.125/20 cm	−4.25/–0.50 × 10	−4.25	1.0	
F3:III:5	24/7	OD	0.06	0.06/20 cm	−4.50/–0.50 × 79	−3.50/–1.25 × 90	0.4	64
		OS	HM/BE	HM/BE	−6.00/–0.50 × 145	HM/BE	HM/BE	

Auto-Ref, autorefraction; Sub-Ref, subjective refraction; BCVA, best corrected visual acuity; PD, pupillary distance; LP, light perception; HM, hand motion; BE, both eyes.

**Table 2:** *CHM* mutations identified in the study.

Subjects	Exon	Variation				SNP137	EVS	ExAC	References
		Nucleotide	Protein	Status	Type				
F1:IV:2	4	c.227_232delinsTGTCATTTCA	p.Q76Lfs*7	Het	Fs	Novel	Novel	Novel	This study
F1:V:2	4	c.227_232delinsTGTCATTTCA	p.Q76Lfs*7	Hemi	Fs	Novel	Novel	Novel	This study
F2:V:5	13	c.1584_1587del TGTT	p.V529Hfs*7	Het	Fs	Rare	Rare	Rare	van den Hurk *et al.*^16^
F3:III:4	6	c.710dupA	p.Y237_S238delinsX	Het	Fs	Novel	Novel	Novel	This study
F3:III:5	6	c.710dupA	p.Y237_S238delinsX	Het	Fs	Novel	Novel	Novel	This study

Het, heterozygous; Hemi, hemizygous; Fs, frameshift.
