# Supplementary material for: Novel CHM mutations identified in Chinese families with Choroideremia
Source: Sci Rep. 2016 Oct 14;6:35360. doi: 10.1038/srep35360 (PMC5064415; doi:10.1038/srep35360)

**Novel *CHM* mutations identified in Chinese families with** **Choroideremia**

Xue-Bi Cai^1†^, Xiu-Feng Huang^1†^, Yi Tong^2^, Qin-Kang Lu^3*^, Zi-Bing Jin^1*^

1. The Eye Hospital of Wenzhou Medical University, The State Key Laboratory Cultivation Base and Key Laboratory of Vision Science, Ministry of Health, Wenzhou 325027, China;

2. Fuzhou Southeastern Eye Hospital, Fuzhou, 350000, China;

3. Department of Ophthalmology, Yinzhou People’s Hospital, Medical School of Ningbo University, Ningbo, 315040, China.

^†^ These authors contributed equally to this work.

***Correspondence**:

E-mail: Dr. Zi-Bing Jin, The Eye Hospital of Wenzhou Medical University, The State Key Laboratory Cultivation Base and Key Laboratory of Vision Science, Ministry of Health, Wenzhou 325027, China. E-mail: jinzb@mail.eye.ac.cn. Tel/fax: +86-577-88067926; Dr. Qin-Kang Lu, Department of Ophthalmology, Yinzhou People’s Hospital, Medical School of Ningbo University, Ningbo, 315040, China. E-mail: luqinkang@163.com.

**Supplementary Figure 1. DNA sequencing profiles of the family members.**


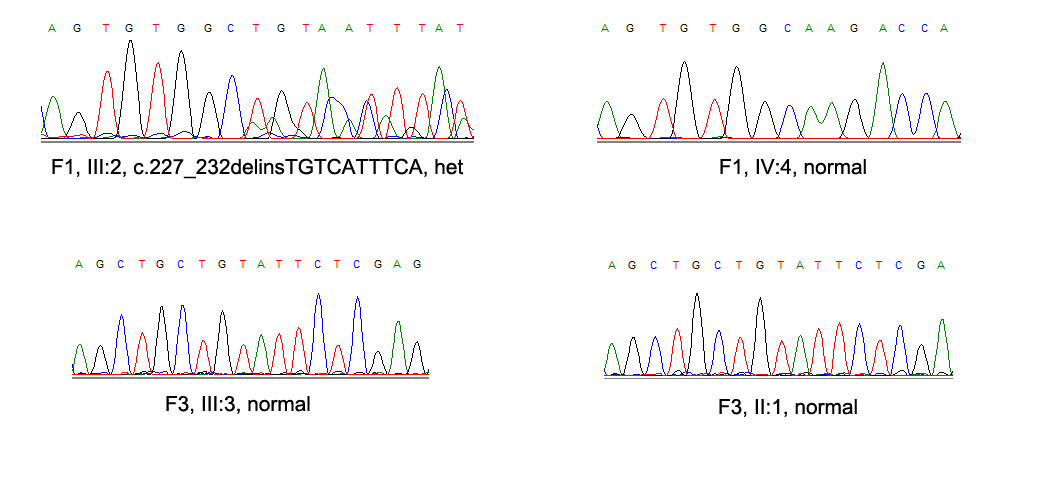

Supplement: Supplementary Information [file srep35360-s1.doc]
